# *miR-199a-3p* suppresses *Vldlr* expression to promote cardiomyocyte proliferation: *Vldlr* and RB1 mediates pro-proliferation effect of *miR-199a-3p* in cardiomyocytes

**DOI:** 10.3724/abbs.2024240

**Published:** 2025-02-10

**Authors:** Rui Jiang, Lijuan Pei, Hongjie Zhang, Fenglian He, Yuhan Min, Xinhang Li, Ke Wei

**Affiliations:** Institute for Regenerative Medicine State Key Laboratory of Cardiology and Medical Innovation Center Shanghai East Hospital Shanghai Key Laboratory of Signaling and Disease Research Frontier Science Center for Stem Cell Research School of Life Sciences and Technology Tongji University Shanghai 200092 China

**Keywords:** cardiomyocyte proliferation, *Vldlr*, *miR-199a-3p*, RB1, cell cycle

## Abstract

The proliferative capacity of cardiomyocytes is limited in adult mammals, and replacing lost tissue following acute ischemic injury is challenging. Previous studies have demonstrated that *miR-199a-3p* can promote cardiomyocyte proliferation, but the exact mechanism by which this occurs remains unclear, although multiple targets of *miR-199a-3p* have been identified. We recently showed that very-low-density-lipoprotein receptor (Vldlr) inhibits cardiomyocyte proliferation, and in this study we aim to test whether *Vldlr* is a functional target gene of *miR-199a-3p*. 3′UTR reporter assays demonstrate that *miR-199a-3p* directly binds to the 3′UTR of *Vldlr* and inhibits its translation. Overexpressing *Vldlr* blunts the pro-proliferative effect of *miR-199a-3p* on cardiomyocytes, suggesting that *Vldlr* is indeed a functional target of *miR-199a-3p*. Mechanistically, Vldlr reduces S807/811 phosphorylation of RB1, and inhibiting CDK4/6 to prevent RB1 phosphorylation can block the pro-proliferative effect of both *Vldlr* knockdown and *miR-199a-3p*, suggesting that RB1 phosphorylation is required for the cardiomyocyte proliferation induced by *miR-199a-3p* and *Vldlr* knockdown. The findings of this study reveal *Vldlr* as a novel functional target of *miR-199a-3p* in cardiomyocytes and identify RB1 as a downstream effector of cardiomyocyte proliferation. The identification of the role of the *miR-199a-3p* - *Vldlr*-RB1 axis in cardiomyocyte proliferation may provide potential therapeutic targets for cardiac regenerative medicine.

## Introduction

The adult mammalian heart lacks regenerative capacity, mainly because cardiomyocytes (CMs) exit the cell cycle during maturation [1]. The loss of cardiomyocytes in cardiac diseases such as myocardial infarction cannot be compensated with new cardiomyocytes, thus compromising the contractility of the remaining myocardium, ultimately leading to heart failure and death when the extent of injury is severe [2]. Numerous studies have identified various regulators of cardiomyocyte proliferation in cultured cardiomyocytes and in small animal models [2]; however, only a few have shown effectiveness in promoting adult cardiomyocyte proliferation in large animal models [ 3, 4]. Elucidating the molecular mechanism by which these factors promote adult cardiomyocyte proliferation in large animals may provide a better understanding of human cardiomyocyte regeneration and cardiac regeneration, as well as potential pharmaceutical targets suitable for human patients. 

MicroRNAs (miRNAs), small noncoding RNAs that have diverse functions, including the promotion of mRNA degradation and the inhibition of mRNA translation, have been demonstrated to play multifaceted roles in cardiac development, disease control and regeneration [5]. A Functional screening revealed that *miR-199a-3p* can induce robust proliferation of neonatal, as well as adult, rodent cardiomyocytes [6]. Importantly, in a swine model, *miR-199a-3p* effectively promoted adult cardiomyocyte proliferation after myocardial infarction while causing lethal arrhythmia, possibly due to uncontrolled dedifferentiation of cardiomyocytes [3]. Given the robust ability of *miR-199a-3p* to induce cardiomyocyte proliferation, there has been a persistent quest to identify its functional targets. Critical transcription factors, including Homer1 and Hopx [ 6– 8]; transcription suppressors, such as NACC2 [9]; regulators of Hippo pathways, including TAOK1 and β-TrCP [10]; and cell membrane receptors, such as Cd151 [11], have been found to be functional targets of *miR-199a-3p* in cardiomyocytes. However, most microRNAs function by targeting multiple mRNAs, and there may be unidentified targets of *miR-199a-3p* that are critical in regulating the CM cell cycle. In addition, the deleterious effects of overexpressing *miR-199a-3p* in adult swine hearts, including tachyarrhythmia, are attributed to possible arrhythmogenic targets and/or coexpressed *miR-199a-5p* [3]. Therefore, identifying novel functional targets of *miR-199a-3p* will not only elucidate the mechanisms by which *miR-199a-3p* regulates cardiomyocyte proliferation but also shed light on the core cell cycle regulation of cardiomyocyte. This may lead to the discovery of novel and specific therapeutic targets for cardiac regenerative medicine, thereby avoiding the off-target effects of *miR-199a-3p*. 

Our previous research on signaling from noncardiomyocytes regulating cardiomyocyte proliferation identified very-low-density-lipoprotein receptor (Vldlr) as a plasma membrane receptor on cardiomyocytes that inhibits proliferation when the suppressive ligand TSP-1 is abundant in the adult heart [12]. Interestingly, both *miR-199a-3p* and Vldlr have been shown to regulate YAP activity in cardiomyocytes [ 10, 12], and *miR-199a-3p*, as well as other pro-proliferative microRNAs, may target multiple components of the Hippo pathway to regulate YAP activity [ 10, 13], thus prompting us to investigate whether *Vldlr* is a functional target of *miR-199a-3p*. 

In this study, we employ neonatal rat ventricular cardiomyocytes (NRVCs) as an *in vitro* model to investigate the regulatory interplay between *Vldlr* and *miR-199a-3p* as a potential novel mechanism of cell cycle regulation in cardiomyocytes, and to identify possible downstream effectors that are core regulators of cardiomyocyte proliferation. 

## Materials and Methods

### Neonatal rat cardiomyocyte isolation and culture

Neonatal rat cardiomyocytes were isolated from the left ventricles on postnatal day 1 Sprague Dawley rats (Shanghai SLAC Laboratory Animal Co., Shanghai, China) via a primary cardiomyocyte isolation kit (AC1002017; Applied Cell, Shanghai, China). The harvested left ventricle tissues were then cut into small pieces with scissors. To remove blood cells, the tissues were washed with ice-cold PBS three times. Subsequently, 4 mL of cell digest solution was added to the fragments, which were then placed in a water bath maintained at 37°C for approximately 5 min to adjust the temperature of the digestion solution. This was done with gentle agitation. The supernatant was subsequently removed via centrifugation at 240 *g*, and a 6 mL mixture was added for digestion at 37°C in a water bath. This solution was then transferred to a new 15-mL conical tube, which was mixed with 6 mL of Dulbecco’s modified Eagle’s medium (DMEM) containing 10% fetal bovine serum (FBS) to halt the enzymatic reaction. This process was repeated four to six times until the heart pieces became very small and white. The digested myocardium mixture was then filtered through a 100-μm nylon mesh filter (CSS013100; Biofil, Guangzhou, China) and centrifuged at 210 *g* for 6 min. The resulting pellet was resuspended in DMEM with 10% FBS, and the cell mixture was transferred to a cell culture dish in a 5% CO _2_ incubator for 90 min. The cardiomyocytes in the supernatant were collected following centrifugation and replated on collagen-coated dishes with 10% DMEM. The cells were then cultured in a 5% CO _2_ incubator for 24 h. On the second day, the culture medium was replaced by DMEM:F12 (1:1) containing 0.25% FBS, 3 mM sodium pyruvate, 0.1 mM vitamin C and 2 mM L-glutamine. All the aforementioned steps were conducted in a sterile cell culture hood. 

Palbociclib (S4482; Selleck, Houston, USA) was added at a final concentration of 0.25 μM.

### Mouse embryonic stem cell-derived cardiomyocyte culture

mESCs ( *Myh6-Puro ^r^*; *Rex-Blast ^r^*) were cultured in differentiation medium as previously described [4]. Puromycin was added at differentiation day 9 for 24 h to purify cardiomyocytes, which were subsequently dissociated and plated as monolayer cardiomyocytes. 

### siRNA-mediated knockdown assay

Cardiomyocytes were transfected with 50 nM *miR-199a-3p* mimic or miR-NC as a negative control (Sangon Biotech, Shanghai, China) in accordance with the protocol outlined in the user guide of Lipofectamine® 2000 Reagent (11668; Invitrogen, Waltham, USA). EdU was added 12 h later, and the cells were fixed and stained 48 h after the addition of EdU. 

siRNA was used to downregulate *Vldlr* expression. Following 48 h of culture, neonatal rat ventricular cardiomyocytes (NRVCs) were transiently transfected with 55 nM siRNA-NC as a negative control or with si- *Vldlr* (Sangon Biotech) via Lipofectamine 2000® Transfection Reagent (Invitrogen) in accordance with the manufacturer’s instructions. 

### AAV9 overexpression assay

To investigate the effects of Vldlr overexpression, NRVCs were transduced with adeno-associated virus serotype 9 (AAV9). We utilized two constructs: AAV9-cTnT- *Vldlr*, which includes the *Vldlr* gene under the control of the cardiac troponin T (cTnT) promoter, and AAV9-cTnT-Blank, which serves as a control. NRVCs were transduced with AAV9-cTnT- *Vldlr* or AAV9-cTnT-Blank for 40 h and recovered in cardiomyocyte medium for 72 h. 

### Immunofluorescence staining

Cardiomyocytes were fixed with 4% paraformaldehyde for 20 min at room temperature, followed by a 5-min wash with 1× PBS. The cells were permeabilized with 0.3% Triton X-100 for 20 min and subsequently blocked with 2% BSA for 2 h at room temperature. After blocking, the cells were incubated with the primary antibody at 4°C overnight. On the following day, the cells were washed three times with 1× PBS for 5 min each and then incubated with the secondary antibody for 1 h at room temperature. The nuclei were stained with DAPI (157574; MP Biomedicals, Santa Ana, USA) across all samples. Cardiomyocytes were identified via antibodies against α-actinin (ab5694; Abcam, Cambridge, UK) or cTnT (ab64623; Abcam), and the phosphorylation of Rb at S807 + S811 was detected via a recombinant anti-Rb (phospho S807 + S811) antibody (ab277774; Abcam), before incubation with respective secondary antibodies. Fluorescence images were acquired via a Axio Vert A1 fluorescence microscope (Zeiss, Oberkochen, Germany) or a Revvity Opera Phenix (Revvity, Waltham, USA) and analyzed with CellProfiler software [14] or Harmony software (Revvity, Waltham, USA). More than three independent replicate experiments were performed for each immunofluorescence stain, and more than 2000 cells were analyzed in each well. 

### EdU incorporation assay

Cell proliferation was assessed by EdU incorporation assay following the protocol provided with the EdU Alexa Fluor™ 488 Imaging Kit (C10637; Thermo Fisher Scientific, Waltham, USA). Briefly, cells were labeled with EdU for either 24 or 48 h prior to fixation. After fixation and permeabilization, the incorporated EdU was detected using the appropriate reaction cocktailas described in the protocol. The cells were incubated with the cocktail for 30 min at room temperature in the dark to prevent photobleaching. Following incubation, the reaction cocktail was removed, and the cells were washed once with 1 mL of 3% BSA in PBS to eliminate any residual reagents. Finally, the cells were imaged using fluorescence microscopy with filters appropriate for the detection of the EdU signal.

### Vector construction and 3′UTR reporter assay

The rat *Vldlr* 3′UTR fragment (5110 bp) was fused to the 3′ end of the *EGFP* gene on the pLenti- *EGFP*-UTR-Blank backbone (BL8246; Applied Biological Materials Inc, Vancouver, Canada) to generate the pLenti- *EGFP* - *Vldlr* 3′UTR construct. A pLenti- *EGFP*- *Vldlr* 3′UTR mutant was also constructed, which contained a mutated binding site of *miR-199a-3p* (5′-ACACTGT-3′ mutated to 5′-TACACTGT-3′, 3187 bp from the start of the 3′UTR) in the *Vldlr* 3′UTR. The AAV9-cTnT- *Vldlr* construct was generated by cloning the mouse *Vldlr* coding sequences from pCMV6- *Vldlr* (Origene, Rockville, USA) and substituting the 3Flag and hYAP fragments in the AAV9-cTnT-3Flag-hYAP (86558; Addgene, Watertown, USA) with the *Vldlr* coding region, as described previously [12]. Wild-type (WT) and mutant (Mut) 3′UTR constructs were co-transfected into HEK293T cells along with miR-199a-3p or negative control miRNA mimics. After 24 h of culture, EGFP signal was imaged using the same parameters with Axio Vert A1 fluorescence microscope. The captured images were analyzed with CellProfiler software, the average EGFP intensity values of each images were calculated, and the average values of each conditions were normalized to the average of the control (WT + control miR) values. 

### Bioinformatics analysis

The RNA sequencing (RNA-Seq) data for si- *Vldlr*-transfected NRVCs and *Vldlr*-overexpressing NRVCs and their respective controls (GSE155658) obtained from our previous research [12] were analyzed. After low-quality reads and adapters were removed, the clean data were mapped via HISAT2, and the transcripts were assembled via StringTie [15]. Differential expression analysis of the gene expression matrix was conducted via the edgeR package in R [16]. RNA-Seq data from deep sequencing of neonatal mouse cardiomyocytes transfected with a *miR-199a-3p* mimic (GSE41538) [6] were integrated with RNA-Seq data of NRVCs transfected with a *miR-199a-3p* mimic (GSE129598) [10] to derive a gene transcript matrix, which was subsequently used for GSEA processing [17]. GSEA software (version 4.1.0; https://www.gsea-msigdb.org/gsea/index.jsp) was used to perform gene set enrichment analysis, specifically using the C6 oncogenic signatures module and the differentially expressed gene lists obtained from the RNA-Seq analysis. 

To identify direct targets of E2F1 and TEAD1, the E2F1 and TEAD1 motif data were downloaded from the JASPAR database [18]. Motif scanning of the 1000 bp upstream regions of genes was performed via the FIMO tool from the MEME Suite [19] to identify the exact coordinates where the E2F1 and TEAD1 motifs are present in gene promoters. BEDTools ( https://bedtools.readthedocs.io/en/latest/index.html) intersect was subsequently used to identify overlapping promoter regions between the E2F1 and TEAD1 motif target BED files. The overlapping regions were then annotated via gene region BED files to identify the E2F1 and TEAD1 target genes. 

### Western blot analysis

Protein samples were extracted via RIPA lysis buffer (PC102; Epizyme Biotech, Shanghai, China) supplemented with protease inhibitors (GRF101; Epizyme Biotech) and phosphatase inhibitors (GRF102; Epizyme Biotech). Protein concentrations were determined via a BCA protein assay kit (P0010S; Beyotime, Shanghai, China). Western blot analysis was conducted in accordance with the standard protocols outlined by Bio-Rad (Hercules, USA). Primary antibodies against GAPDH (ab9485) and phospho-S807 + S811 Rb (ab277774) from Abcam, as well as Rb1 (P010747) from Epizyme, were used, each at a 1:1000 dilution. Secondary antibodies, including goat anti-mouse (AF922) and goat anti-rabbit (AH620), were obtained from Sungene Biotech (Tianjin, China) and applied at a dilution of 1:5000. The results were visualized via the ChemiScope 6000 Exp system (CLinx, Shanghai, China).

### Quantitative real-time PCR

Total RNA was extracted via TRIzol reagent (Invitrogen). The complementary DNA was synthesized via the PrimeScript™ RT Reagent Kit (RR047A; Takara, Kusatsu, Japan) according to the manufacturer’s instructions. Quantitative real-time polymerase chain reaction (qRT-PCR) was carried out by using SYBR Green Premix Ex Taq (AK8806; Takara) on a CFX384 Real-Time System (C1000; Bio-Rad). The cDNA synthesis and qRT-PCR of miRNA were performed following the user manual of the Mir-X™ miRNA First-Strand Synthesis and qRT-PCR Kit (638313; Takara). All primer sequences are shown in Table 1.

**Table:** **Table 1** Sequences of the primers used for qRT-PCR

Gene/miRNA	Forward (5′→3′)	Reverse (5′→3′)
*Aurka*	AAGCAAAGCAAGTTCATCCTGG	TGTTCCAAGGGGCGCGTATTC
*Cdkt*	AGAAGGTACTTACGGTGTGGT	GAGAGATTTCCCGAATTGCAGT
*Cdk2*	CCTGCTTATCAATGCAGAGGG	TGCGGGTCACCATTTCAGC
*Gapdh*	GACATGCCGCCTGGAGAAAC	AGCCCAGGATGCCCTTTAG
*Mki67*	ATCATTGACCGCTCCTTTAGGT	GCTCGCCGTGATGATTCCT
*Vldlr*	AAAATGGCGGTGTGACGGAGA	TCAACACAGTCTCGGATGCCA
*miR-199a-3p*	ACAGUAGUCUGCACAUUGGUUA	
*miR-NC*	UUGUACUACACAAAAGUACUG	

### Statistical analysis

Student’s *t* test and ANOVA were used to determine the statistical significance for the data comparisons. Data are expressed as the mean ± standard error of the mean (SEM). A *P* value of less than 0.05 is considered statistically significant 

## Results

### *Vldlr* is a direct target of *miR-199a-3p*

**Figure 1:**
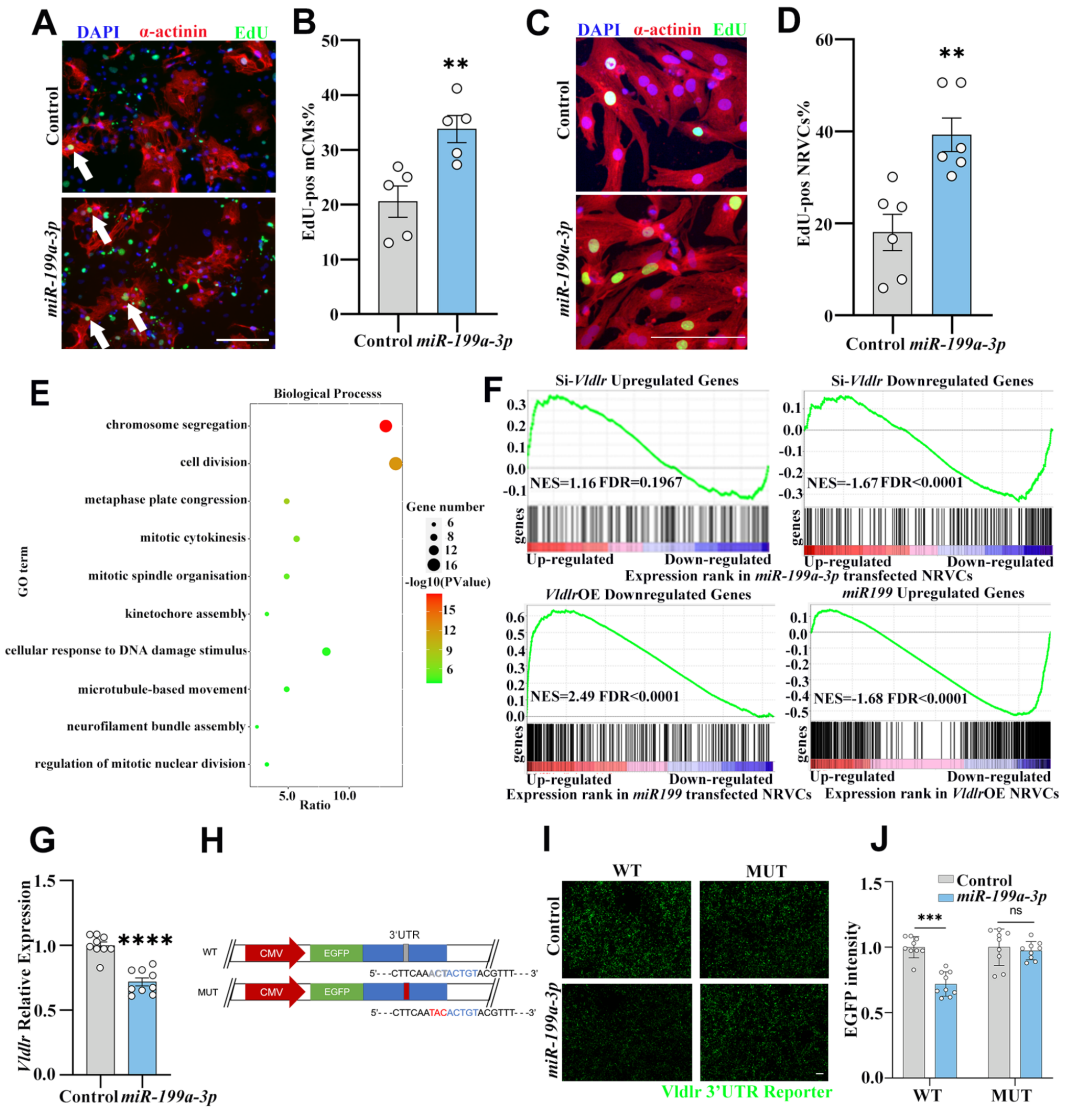
Figure 1 *Vldlr* is a direct target of *miR-199a-3p* (A) Immunofluorescence staining for DAPI (blue), α-actinin (red) and EdU (green) in mCMs transfected with the miR-199a-3p mimic or control. The arrows indicate EdU-positive cardiomyocytes. (B) Percentage of EdU-positive cardiomyocytes in mCMs transfected with the miR-199a-3p mimic or the control. n is the well number, n = 5. (C) Immunofluorescence staining for DAPI (blue), α-actinin (red) and EdU (green) in NRVCs transfected with the miR-199a-3p mimic or the control. (D) Percentage of EdU-positive cardiomyocytes in NRVCs transfected with the miR-199a-3p mimic or the control. n is the well number, n = 6. (E) Gene Ontology (GO) term analysis of the biological processes of the overlapping genes that were upregulated in NRVCs transfected with the miR-199a-3p mimic and downregulated in NRVCs overexpressing Vldlr. (F) Gene set enrichment analysis (GSEA) of upregulated and downregulated genes upon Vldlr knockdown in RNA-Seq results from NRVCs transfected with the miR-199a-3p mimic vs the control. GSEA of the genes downregulated by overexpressing Vldlr in RNA-Seq results from NRVCs transfected with the miR-199a-3p mimic vs the control, and genes upregulated by miR-199a-3p mimic transfection in RNA-Seq results from NRVCs overexpressing Vldlr vs the control. (G) qRT-PCR results of Vldlr expression in NRVCs transfected with the miR-199a-3p mimic or the control. n = 9. (H) Schematic of the EGFP reporter fused with the Vldlr 3′UTR with wild-type (gray) and mutated (red) miR-199a-3p binding motifs. (I) EGFP fluorescence in HEK293T cells cotransfected with 50 nM miR-199a-3p mimic or control with 20 ng of the wild-type or mutant pLenti-EGFP-Vldlr 3′UTR construct. (J) Quantification of EGFP fluorescence in HEK293T cells transfected with wild-type or mutant pLenti-EGFP- Vldlr 3′UTR reporters with or without the miR-199a-3p mimic. n is the field number, n = 9. Scale bar: 100 μm. Data are expressed as the mean ± SEM. **P < 0.01, *** P < 0.001, ****P < 0.0001; ns, not significant.

To identify potential novel targets of *miR-199a-3p* in cardiomyocytes, we first sought to confirm the effect of *miR-199a-3p* on cardiomyocytes proliferation in both mouse embryonic stem cells-derived cardiomyocytes (mCMs) and neonatal rat ventricular cardiomyocytes (NRVCs). *miR-199a-3p* expression was significantly upregulated by 2.3-fold in the *miR-199a-3p* mimic-transfected NRVCs compared with the control ( Supplementary Figure S1A), and cardiomyocyte areas were not significantly altered by the *miR-199a-3p* mimic ( Supplementary Figure S1B). Consistent with previous reports [ 6 , 10], we found that the *miR-199a-3p* mimic significantly increased EdU incorporation in both mCMs and NRVCs ( Figure 1A–D). Our previous study revealed that very-low-density-lipoprotein receptor (Vldlr) plays an important role in the cell cycle regulation of neonatal cardiomyocytes by consolidating the effects of the pro-proliferation ligand Reelin and the anti-proliferation ligand TSP-1 [12], and it suppresses adult cardiomyocyte proliferation, as the suppressive TSP-1 is dominant in the adult heart [12]. We previously showed that knocking down and overexpressing *Vldlr* promotes and suppresses cardiomyocyte proliferation, respectively [12]. Therefore, we wondered whether *Vldlr* is a direct target of *miR-199a-3p*. We first compared the transcriptomic changes upon *miR-199a-3p* mimic transfection and upon *Vldlr* knockdown and overexpression ( Supplementary Figure S2A,B). Gene Ontology (GO) analysis of the biological processes of the genes whose expressions were upregulated with *miR-199a-3p* mimic transfection (GSE41538, GSE129598) [ 6, 10] and downregulated upon *Vldlr* overexpression (GSE155658) [12] revealed a significant enrichment of proliferation-related genes ( Figure 1E and Supplementary Table S1), confirming that *miR-199a-3p* and Vldlr have opposite effects on cardiomyocyte proliferation. Gene set enrichment analysis (GSEA) [17] revealed that genes upregulated upon *Vldlr* knockdown were significantly upregulated in NRVCs transfected with the *miR-199a-3p* mimic, whereas genes downregulated by *Vldlr* knockdown were also significantly downregulated in NRVCs transfected with the *miR-199a-3p* mimic ( Figure 1F). Furthermore, genes downregulated upon *Vldlr* overexpression were significantly upregulated in NRVCs transfected with the *miR-199a-3p* mimic and vice versa ( Figure 1F), suggesting that *Vldlr* and *miR-199a-3p* may regulate similar downstream effectors ( Figure 1F). In addition, the expression of *Vldlr* upon *miR-199a-3p* mimic transfection in both mouse and rat cardiomyocytes (GSE41538, GSE129598) [ 6, 10] was downregulated ( Supplementary Figure S3A). Subsequent qRT-PCR analysis revealed significant downregulation of *Vldlr* in NRVCs upon *miR-199a-3p* mimic transfection ( Figure 1G), suggesting that *Vldlr* could be a direct functional target of *miR-199a-3p*. Since miRNAs inhibit gene expression mainly by binding to the 3′-untranslated region (3′UTR) of target mRNAs [20], we developed a fluorescent reporter by fusing the rat *Vldlr* 3′UTR fragment to the 3′ end of the *EGFP* gene ( Figure 1H), to investigate the direct targeting of the *Vldlr* 3′UTR by *miR-199a-3p*. According to miRbase, there is one possible binding site for *miR-199a-3p* within the *Vldlr* 3′UTR ( Figure 1H) [21]. To assess whether this binding site mediates the effect of *miR-199a-3p* on the *Vldlr* 3′UTR, we mutated three bases within the binding site to create a mutant EGFP reporter construct ( Figure 1H). A significant decrease in EGFP intensity was observed in cells transfected with the wild-type *Vldlr* 3′UTR reporter after *miR-199a-3p* mimic transfection, whereas this reduction was not observed in cells transfected with the mutant reporter lacking the binding site for *miR-199a-3p* ( Figure 1I,J). These findings indicate that *Vldlr* is a direct target of *miR-199a-3p* in cardiomyocytes.

### *miR-199a-3p* promotes cardiomyocyte proliferation by suppressing *Vldlr*

**Figure 2:**
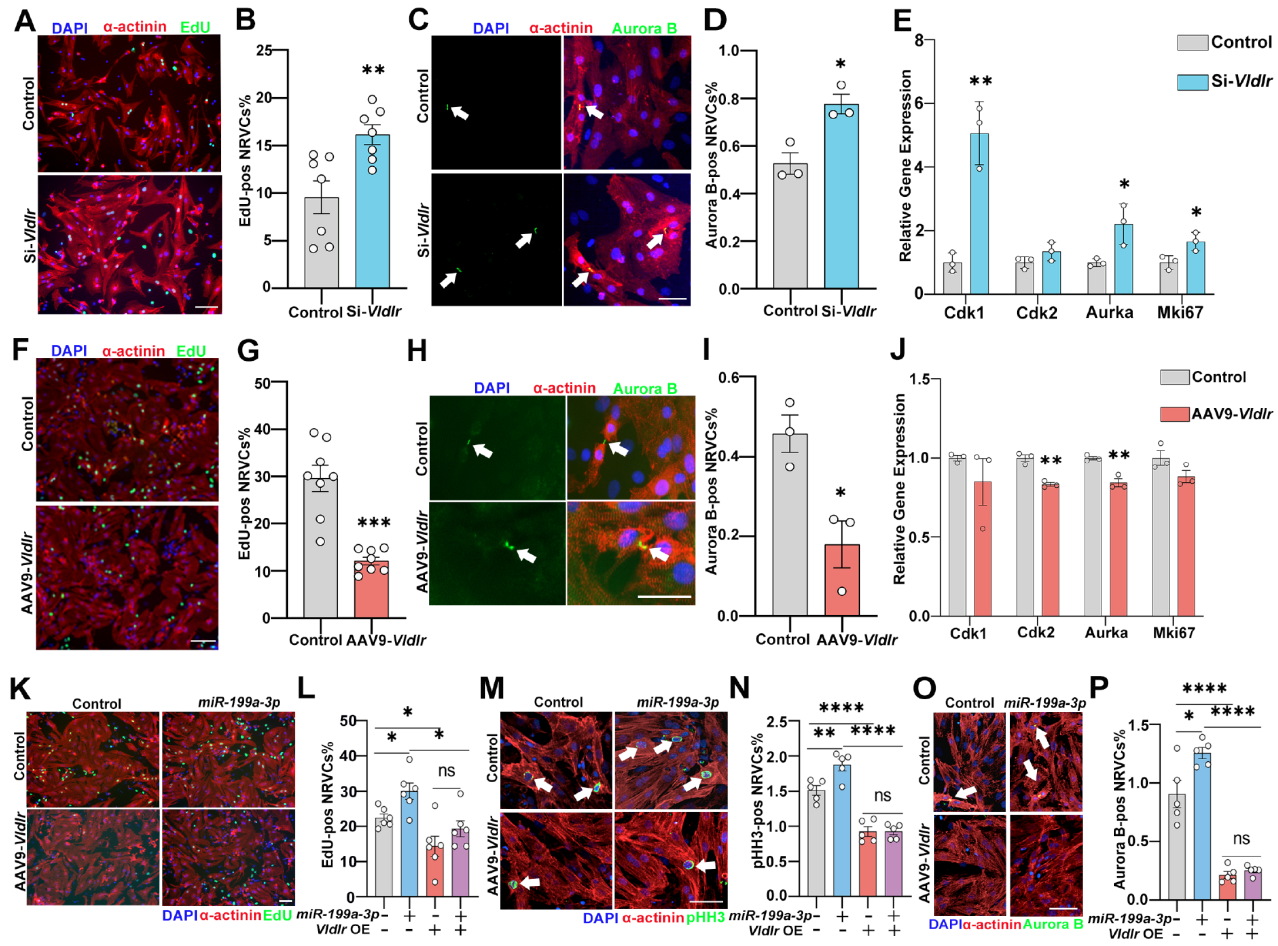
Figure 2 *miR-199a-3p* promotes cardiomyocyte proliferation by suppressing *Vldlr* (A) Immunofluorescence staining for DAPI (blue), α-actinin (red) and EdU (green) in si-Vldlr- and control-transfected NRVCs. Scale bar: 200 μm. (B) Percentage of EdU-positive cardiomyocytes in NRVCs transfected with si-Vldlr vs the control. n is the well number, n = 7. (C) Immunofluorescence staining for DAPI (blue), α-actinin (red) and Aurora B (green) in si-Vldlr- and control-transfected NRVCs. Scale bar: 100 μm. (D) Percentage of Aurora B-positive cleavage furrows between dividing cardiomyocytes in NRVCs transfected with si-Vldlr vs the control. n is the well number, n = 3. (E) qRT-PCR analysis of the expressions of cell cycle-related genes in NRVCs transfected with si-Vldlr vs the control. n = 3. (F) Immunofluorescence staining for DAPI (blue), α-actinin (red) and EdU (green) in si-Vldlr- and control-transfected NRVCs. Scale bar: 200 μm. (G) Percentage of EdU-positive cardiomyocytes in NRVCs transfected with si-Vldlr vs the control. n is the well number, n = 8. (H) Immunofluorescence staining for DAPI (blue), α-actinin (red) and Aurora B (green) in AAV9-Vldlr and control-transduced NRVCs. Scale bar: 50 μm. (I) Percentages of Aurora B-positive cleavage furrows between dividing cardiomyocytes in NRVCs transduced with AAV9- Vldlr and control cardiomyocytes. n is the well number, n = 3. (J) qRT-PCR analysis of the expressions of cell cycle-related genes in NRVCs transfected with AAV9-Vldlr vs the control. n = 3. (K) Immunofluorescence staining for DAPI (blue), α-actinin (red) and EdU (green) in NRVCs transfected with the miR-199a-3p mimic and transduced with AAV9-Vldlr or the corresponding controls. Scale bar: 200 μm. (L) Percentage of EdU-positive cardiomyocytes in NRVCs transfected with the miR-199a-3p mimic and transduced with AAV9-Vldlr or the corresponding controls. n is the well number, n = 6. (M) Immunofluorescence staining for DAPI (blue), α-actinin (red) and pHH3 (green) in NRVCs transfected with the miR-199a-3p mimic and transduced with AAV9-Vldlr and the corresponding controls. Scale bar: 50 μm. (N) Percentage of pHH3-positive cardiomyocytes in NRVCs transfected with the miR-199a-3p mimic and transduced with AAV9- Vldlr or the corresponding controls. n is the well number, n = 5. (O) Immunofluorescence staining for DAPI (blue), α-actinin (red) and Aurora B (green) in NRVCs transfected with the miR-199a-3p mimic and transduced with AAV9-Vldlr or the corresponding controls. Scale bar: 50 μm. (P) Percentage of Aurora B-positive cardiomyocytes in NRVCs transfected with the miR-199a-3p mimic and transduced with AAV9-Vldlr or the corresponding controls. n is the well number, n = 5. The white arrows indicate pHH3-positive cardiomyocytes (M) and Aurora B-positive cleavage furrows between dividing cardiomyocytes (C,H,O). Data are expressed as the mean ± SEM. *P < 0.05, **P < 0.01, ***P < 0.001, ****P < 0.0001; ns, not significant.

To investigate whether targeting *Vldlr* mediates the pro-proliferative effect of *miR-199a-3p* in cardiomyocytes, we first validated the effects of knocking down and overexpressing *Vldlr* on cardiomyocyte proliferation. Consistent with our previous study [12], knocking down *Vldlr* with siRNA in NRVCs led to a significant increase in EdU incorporation ( Figure 2A,B) and a greater proportion of cardiomyocytes with Aurora kinase B (Aurora B) localized in the cleavage furrow between dividing cells during cytokinesis ( Figure 2C,D). In addition, significant upregulation of the cell cycle genes *Cdk1* , *Aurka*, and *Mki67* was observed upon *Vldlr* knockdown ( Figure 2E). On the other hand, overexpressing *Vldlr* in NRVCs with AAV9-cTnT- *Vldlr* transduction led to a significant reduction in EdU incorporation ( Figure 2F,G), a notable decrease in the ratio of cardiomyocytes positive for Aurora B in the cleavage furrow ( Figure 2H,I), and decreased expression of cell cycle genes ( Figure 2J). No significant change in cardiomyocyte area was observed with *Vldlr* knockdown or overexpression ( Supplementary Figure S4A,B). To assess whether *miR-199a-3p* functions by inhibiting *Vldlr* to promote cardiomyocyte proliferation, NRVCs were cotransfected with a *miR-199a-3p* mimic with or without AAV9-cTnT- *Vldlr*. We found that *miR-199a-3p* increases, and AAV9-cTnT- *Vldlr* suppresses, the proliferation of NRVCs, as expected. Importantly, overexpressing *Vldlr* blocked the pro-proliferative effect of *miR-199a-3p* ( Figure 2K–P), suggesting that *Vldlr* is a functional target of *miR-199a-3p* and that *miR-199a-3p* promotes cardiomyocyte proliferation at least partially by inhibiting *Vldlr* expression. As other targets of *miR-199a-3p* have been reported to play a role in cardiomyocyte proliferation, we examined whether modulating *Vldlr* expression affects the expression of other *miR-199a-3p* targets. Analysis of RNA-seq results of NRVCs with *Vldlr* overexpression and knockdown (GSE155658) revealed no significant changes in the mRNA levels of *miR-199a-3p* target genes ( *Cd151* [11], *NACC2* [9], *Pak4* [22], *Rheb* [23], *Homer1* [6] , *Hopx* [6], *BTRC* [10] and *TAOK1* [10]) upon *Vldlr* manipulation ( Supplementary Figure S5A), suggesting that *Vldlr* may act as an independent target of *miR-199a-3p* that does not function as an upstream regulator of other known targets. Taken together, our findings confirmed the crucial role of *Vldlr* as a suppressor of cardiomyocyte proliferation and demonstrated that *miR-199a-3p* promotes cardiomyocyte proliferation by suppressing *Vldlr* expression.

### Vldlr regulates RB1 phosphorylation in cardiomyocytes

**Figure 3:**
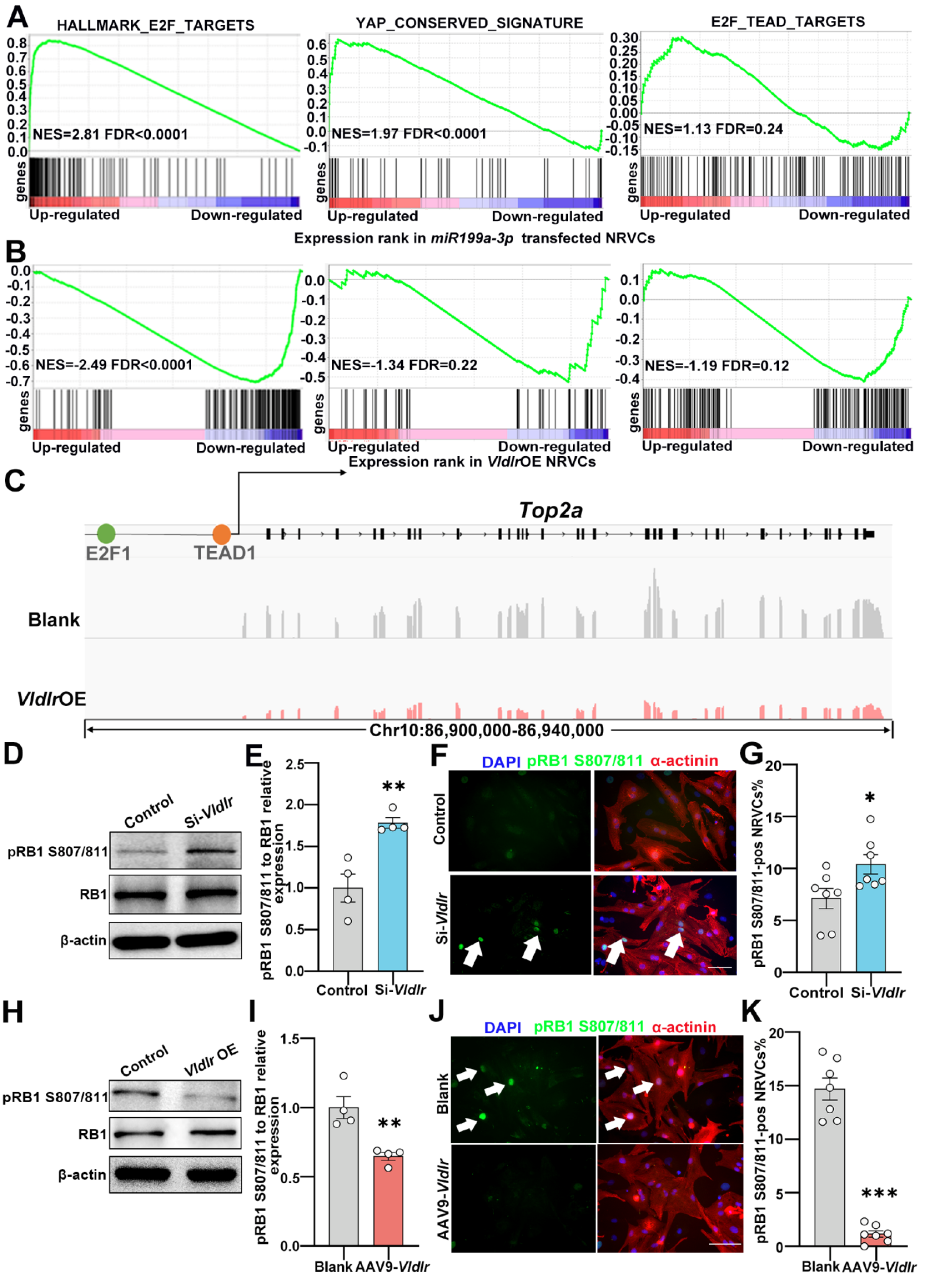
Figure 3 Vldlr regulates RB1 activity in cardiomyocytes (A) Gene set enrichment analysis of E2F target genes, conserved YAP target genes (C6 oncogenic gene sets from MSigDB) and common target genes of E2F and YAP in miR-199a-3p mimic-transfected NRVCs. (B) Gene set enrichment analysis of E2F target genes, conserved YAP target genes (C6 oncogenic gene sets from MSigDB) and common target genes of E2F and YAP in Vldlr-overexpressing NRVCs. (C) The presence of E2F1 and TEAD1 binding motifs on the promoter of the Top2a gene, a representative proliferation gene known to be a target gene of both E2F and YAP, and the RNA-Seq results of Top2a in NRVCs with and without Vldlr overexpression. (D) Western blots of pRB1 S807/811, RB1, and β-actin in NRVCs transfected with si- Vldlr vs the control. (E) Quantification of pRB1 S807/811 levels relative to RB1 protein levels in NRVCs transfected with si-Vldlr vs the control. n = 4. (F) Immunofluorescence staining for DAPI (blue), α-actinin (red) and pRB1 S807/811 (green) in NRVCs transfected with si-Vldlr vs the control. (G) Percentage of pRB1 S807/811-positive cardiomyocytes in NRVCs transfected with si-Vldlr vs the control. n is the well number, n = 7. (H) Western blots of pRB1 S807/811, RB1, and β-actin in NRVCs overexpressing Vldlr vs the control. (I) Quantification of pRB1 S807/811 levels relative to RB1 protein levels in NRVCs overexpressing Vldlr vs the control. n = 4. (J) Immunofluorescence staining for DAPI (blue), α-actinin (red) and pRB1 S807/811 (green) in NRVCs overexpressing Vldlr vs the control. (K) Percentage of pRB1 S807/811-positive cardiomyocytes in NRVCs overexpressing Vldlr vs the control. n is the well number, n = 7. Scale bar: 50 μm. Arrows indicate pRB1 S807/811-positive cardiomyocytes. Data are expressed as the mean ± SEM. *P < 0.05, **P < 0.01, ***P < 0.001.

Both *miR-199a-3p* and *Vldlr* have been shown to regulate cardiomyocyte proliferation through the transcription factor YAP [ 6, 12], with the former targeting genes in the Hippo pathway [6] and the latter signaling through small GTPases such as Rac1 [12]. YAP has been shown to coordinate with the transcription factor E2F to regulate the transcriptional program of cell cycle genes [24], and direct transcriptional targets of YAP and E2F significantly overlap with the binding sites for both YAP/TEAD and E2F around their transcriptional start sites [25]. The activity of E2F is tightly regulated by the retinoblastoma protein (RB1) [26], as phosphorylation of RB1 releases E2F from its sequestration and activates the transcriptional activity of E2F, leading to increased expression of E2F targets, including cell cycle genes [27]. To investigate whether E2F/RB1 activity is involved in cardiomyocyte proliferation regulated by *miR-199a-3p* and *Vldlr*, we performed gene set enrichment analysis (GSEA) and observed an upregulation of E2F target genes, as well as YAP target genes, in NRVCs transfected with the *miR-199a-3p* mimic ( Figure 3A) and a significant decrease in E2F target genes and YAP target genes in NRVCs overexpressing *Vldlr* ( Figure 3B). Consistent with previous reports [25], the genes with both E2F and TEAD motifs in their promoters were also significantly upregulated upon *miR-199a-3p* mimic transfection and downregulated upon *Vldlr* overexpression ( Figure 3A,B). For example, *Top2a*, a well-known gene required for the cell cycle [28], has both E2F1 (E2F transcription factor 1) and TEAD1 binding sites in its promoter, and its expression is significantly suppressed upon *Vldlr* overexpression ( Figure 3C). These results suggest that E2F activity is indeed regulated by *miR-199a-3p* and *Vldlr*. RB1 activity is regulated by a series of phosphorylation events on multiple serine and threonine residues, initially by cyclin D/cyclin-dependent kinase 4 and subsequently by cyclin E/CDK2 complexes [29] during the cell cycle, and the phosphorylation of S807/811 on RB1 by CDK4/6 [29] is a well-characterized crucial activating regulatory mechanism of E2F activity [ 27, 30]. Therefore, we examined the level of Ser807/811 phosphorylation of RB1 via western blot analysis and immunofluorescence staining and found that *Vldlr* knockdown increased, whereas *Vldlr* overexpression suppressed, S807/811 phosphorylation of RB1 ( Figure 3D–K), suggesting that *Vldlr* suppresses RB1 phosphorylation in cardiomyocytes.

### *miR-199-3p* promotes RB1 phosphorylation through *Vldlr* suppression in cardiomyocytes 

**Figure 4:**
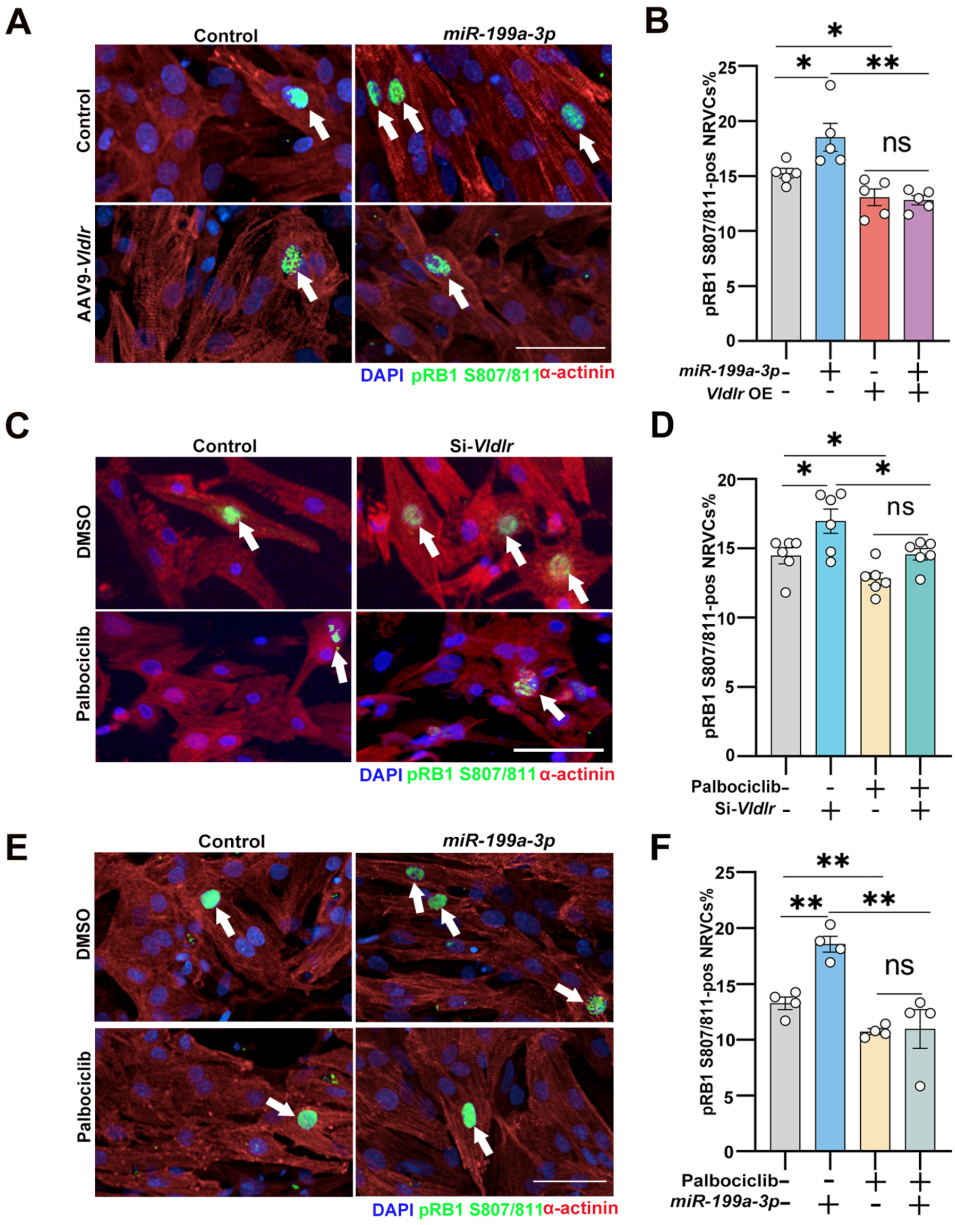
Figure 4 *miR-199a-3p* promotes RB1 phosphorylation through Vldlr suppression in cardiomyocytes (A) Immunofluorescence staining for DAPI (blue), α-actinin (red) and pRB1 S807/811 (green) in NRVCs transfected with the miR-199a-3p mimic and transduced with AAV9-Vldlr or the corresponding controls. (B) Percentage of pRB1 S807/811-positive cardiomyocytes in NRVCs transfected with the miR-199a-3p mimic and transduced with AAV9-Vldlr or the corresponding controls. n is the well number, n = 5. (C) Immunofluorescence staining for DAPI (blue), α-actinin (red) and pRB1 S807/811 (green) in NRVCs transfected with si-Vldlr or si-control, with or without palbociclib. (D) Percentage of pRB1 S807/811-positive cardiomyocytes in NRVCs transfected with si-Vldlr or the control, with or without palbociclib. n is the well number, n = 6. (E) Immunofluorescence staining for DAPI (blue), α actinin (red) and pRB1 S807/811 (green) in NRVCs transfected with the miR-199a-3p mimic or control, with or without palbociclib. (F) Percentage of pRB1 S807/811-positive cardiomyocytes in NRVCs transfected with the miR-199a-3p mimic or the control, with or without palbociclib. n is the well number, n = 4. Scale bar: 50 μm. Arrows indicate pRB1 S807/811-positive cardiomyocytes (A,C,E). Data are expressed as the mean ± SEM. *P < 0.05, **P < 0.01, ns, not significant.

Since we established that *miR-199a-3p* suppresses *Vldlr* expression ( Figure 1) and that Vldlr suppresses RB1 phosphorylation in cardiomyocytes, we sought to examine whether *miR-199a-3p* regulates RB1 phosphorylation via *Vldlr*. We performed immunofluorescence staining of pRB1 S807/811 in NRVCs transfected with *miR-199a-3p* with or without AAV9- *Vldlr* transduction. Quantification of the immunofluorescence staining of pRB1 S807/811 revealed that *Vldlr* overexpression blunted the increase in pRB1 S807/811-positive cardiomyocytes induced by *miR-199a-3p* ( Figure 4A,B). This observation strongly suggests that *miR-199a-3p* increases RB1 phosphorylation through the suppression of *Vldlr*. As CDK4/6 are known kinases that phosphorylate S807/811 on RB1 [29], to validate whether CDK4/6 activity indeed mediates the effects of *Vldlr* knockdown as well as *miR-199a-3p* overexpression on RB1 phosphorylation, NRVCs were treated with palbociclib, an inhibitor of CDK4/6 [29], under conditions of *Vldlr* knockdown, as well as in the presence of *miR-199a-3p* overexpression. Immunofluorescence staining of pRB1 S807/811 revealed a significant increase in the ratio of pRB1 S807/811-positive cardiomyocytes upon *Vldlr* knockdown, which was blunted by palbociclib treatment, suggesting that RB1 phosphorylation by CDK4/6 is regulated by *Vldlr* ( Figure 4C,D). Consistently, the ratio of pRB1 S807/811-positive cardiomyocytes was significantly increased upon *miR-199a-3p* mimic transfection, which was blunted by palbociclib treatment, suggesting that RB1 phosphorylation by CDK4/6 is downstream of *miR-199a-3p* ( Figure 4E,F). Taken together, these findings suggest that *miR-199-3p* promotes RB1 phosphorylation via CDK4/6 through the suppression of *Vldlr*.

### Knockdown of *mir-199a-3p* and *Vldlr* promotes cardiomyocyte proliferation by increasing RB1 phosphorylation 

**Figure 5:**
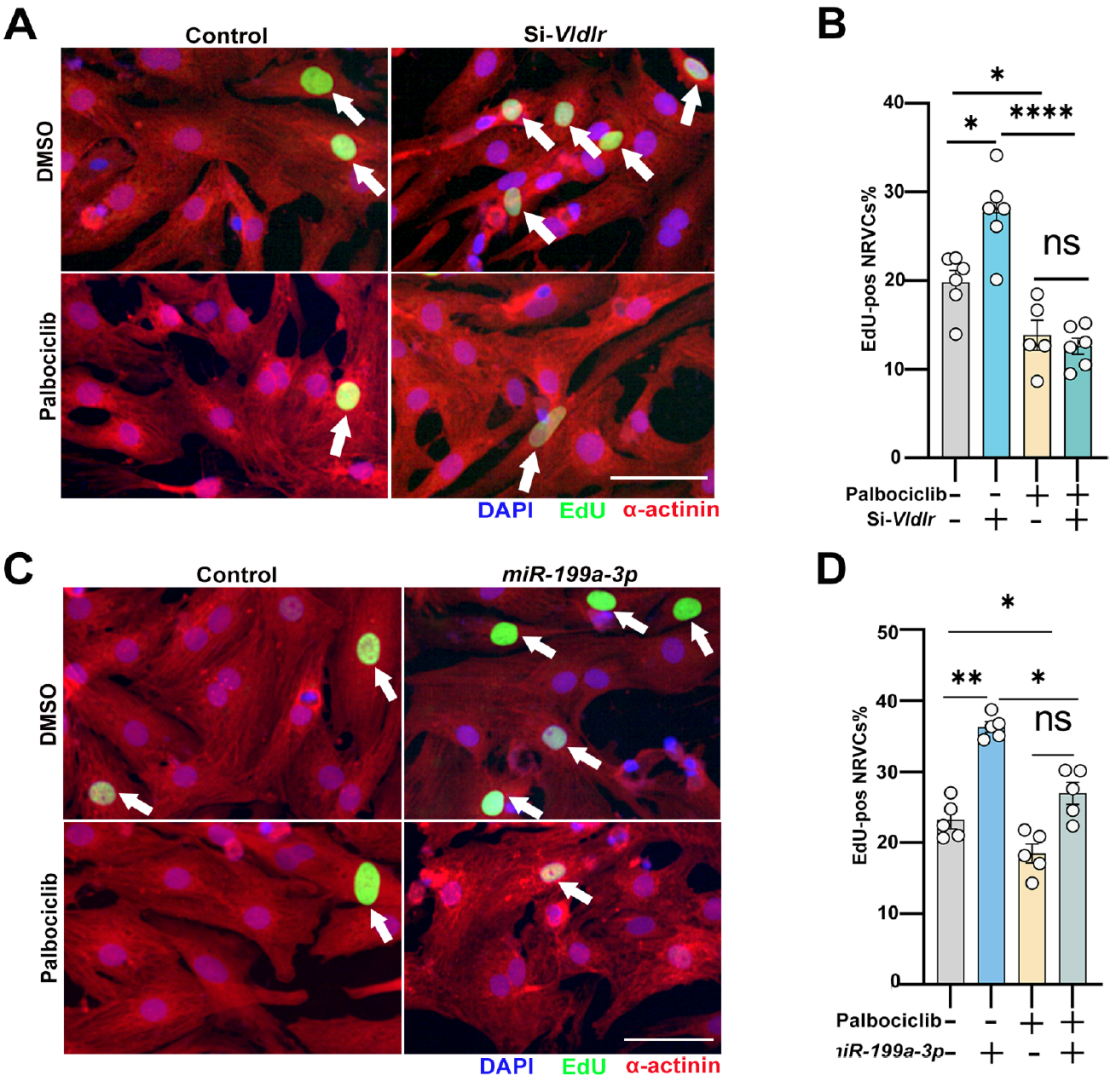
Figure 5 Knockdown of *miR-199a-3p* and *Vldlr* promotes CM proliferation via RB1 phosphorylation (A) Immunofluorescence staining for DAPI (blue), α-actinin (red) and EdU (green) in NRVCs transfected with si-Vldlr or the control, with or without palbociclib. (B) Percentage of EdU-positive cardiomyocytes in NRVCs transfected with si- Vldlr or the control, with or without palbociclib. n is the well number, n = 6. (C) Immunofluorescence staining for DAPI (blue), α-actinin (red) and EdU (green) in NRVCs transfected with the miR-199a-3p mimic or the control, with or without palbociclib. (D) Percentage of EdU-positive cardiomyocytes in NRVCs transfected with the miR-199a-3p mimic or the control, with or without palbociclib. n is the well number, n = 5. Scale bar: 50 μm. Arrows indicate EdU-positive cardiomyocytes (A,C). Data are expressed as the mean ± SEM. *P < 0.05, **P < 0.01, **** P < 0.0001; ns, not significant.

Since we have demonstrated that *miR-199a-3p* directly targets *Vldlr* to promote cardiomyocyte proliferation and regulates RB1 phosphorylation through *Vldlr*, we next sought to explore whether RB1 phosphorylation by CDK4/6 mediates the effects of *miR-199a-3p* and *Vldlr* knockdown on cardiomyocyte proliferation. We knocked down *Vldlr* and transfected a *miR-199a-3p* mimic into NRVCs with or without palbociclib treatment and found that palbociclib blunted the pro-proliferative effects of both *Vldlr* knockdown ( Figure 5A,B) and *miR-199a-3p* mimic transfection ( Figure 5C,D). Combined with the results that *miR-199a-3p* suppresses *Vldlr* expression to promote cardiomyocyte proliferation ( Figures 1 and 2) and regulates RB1 phosphorylation through *Vldlr* ( Figures 3 and 4), these results suggest that the phosphorylation of RB1 at serine 807/811 by CDK4/6 is functionally downstream of *miR-199a-3p* and *Vldlr* suppression, leading to enhanced cell cycle activity in cardiomyocytes. Hence, our results identified RB1 as a downstream effector of *Vldlr* and *miR-199a-3p* in the regulation of cardiomyocyte proliferation.

## Discussion

MicroRNAs have emerged as pivotal regulators of cardiac development and cardiomyocyte proliferation through their ability to bind to mRNAs and control gene expression [5]. In addition to *miR-199a-3p*, an increasing number of microRNAs, such as *miR-17-92* [31], *miR-99*/ *100* [32], Let-7a/c [33], and *miR-302* [34], have been identified as regulators of cardiomyocyte proliferation. Nevertheless, only *miR-199a-3p* has been shown to be effective at promoting cardiomyocyte proliferation in large animal models [3], despite a lethal side effect of arrythmia [3]. The number of target mRNAs that one microRNA can inhibit has always been a hurdle for the therapeutic application of microRNAs [5]. However, microRNAs that regulate critical biological processes, such as cardiomyocyte proliferation, may also target multiple components of a common core pathway, such as the Hippo-Yap pathway [ 10, 13]. Therefore, identifying key functional targets of *miR-199a-3p* will not only provide potential drug targets for regenerative medicine but also prevent unwanted side effects of microRNA therapies. 

In addition to its role as a lipoprotein receptor [35] regulating cholesterol and fatty acid metabolism [36] , *Vldlr* has been shown to interact with various extracellular ligands, such as Reelin [37] and TSP-1 [38], and regulate a variety of cellular behaviors, including cell migration and proliferation [39]. We recently reported that *Vldlr* on the plasma membrane of cardiomyocytes consolidates pro- and antiproliferation signals, such as Reelin and TSP-1, respectively, in the microenvironment of cardiomyocytes [12]. In the adult heart, *Vldlr* functions to inhibit cardiomyocyte proliferation, as the suppressive TSP-1 signal is dominant [12]. Interestingly, we found that *Vldlr* acts through Rac1 to regulate YAP activity [12], which is core to cardiomyocyte proliferation [13]. Given the similarity of regulatory networks affecting cardiomyocyte proliferation downstream of *miR-199a-3p* and *Vldlr* [ 10, 12], we tested whether *Vldlr* is a direct and functional target of *miR-199a-3p*. We confirmed through 3’UTR reporter assays as well as functional rescue assays that *Vldlr* is a direct target of *miR-199a-3p* in regulating cardiomyocyte proliferation. Importantly, a mechanistic study revealed that RB1 phosphorylation and subsequent E2F activity are regulated by *miR-199a-3p* and Vldlr and that blocking RB1 phosphorylation with a CDK4/6 inhibitor can blunt the pro-proliferative effect of *miR-199a-3p* and *Vldlr* knockdown. Our results identified a novel functional target of *miR-199a-3p* and revealed RB1 as a critical regulator of cardiomyocyte proliferation downstream of *miR-199a-3p* and Vldlr, which may provide new therapeutic targets for cardiac regeneration. 

RB transcriptional corepressor 1 (RB1), a major member of the RB family of proteins, plays a vital role in the regulation of cell cycle gene expression [30], and RB1 phosphorylation plays an important regulatory role in controlling the transcriptional activity of E2F transcription factors and the expressions of their target genes [ 27, 30]. Importantly, RB family members suppress cardiomyocyte proliferation by inhibiting the expressions of E2F targets [40]. Our data demonstrated that *miR-199a-3p* and Vldlr act upstream of RB1 to regulate CM proliferation, suggesting a new layer of RB1 regulation in cardiomyocytes. It remains unclear how Vldlr regulates RB1 phosphorylation and whether this regulation is dependent on known downstream signaling pathways of Vldlr, such as Rac1 and YAP [12], which warrants further investigation. In addition, research on animal models of myocardial infarction is necessary to ascertain whether the mechanism identified in our current study in isolated primary cardiomyocytes remains functional in cardiomyocytes within the hearts of live animals. This is because adult cardiomyocytes *in vivo* exhibit notable differences from the neonatal cardiomyocytes utilized in our study. 

In summary, we found that *miR-199a-3p* directly inhibits the expression of the *Vldlr* gene to promote cardiomyocyte proliferation. Vldlr inhibits the phosphorylation of RB1 via CDK4/6, and blocking RB1 phosphorylation blunts the effect of *miR-199a-3p* as well as *Vldlr* knockdown, thus revealing RB1 as a key regulator of cardiomyocyte proliferation downstream of *miR-199a-3p* and *Vldlr*. 

## Supporting information

465TabS1

465FigS1-5

## Supplementary Data

Supplementary data is available at *Acta Biochimica et Biophysica Sinica* online.
