# Supplementary material for: miR-199a-3p suppresses Vldlr expression to promote cardiomyocyte proliferation : Vldlr and RB1 mediates pro-proliferation effect of miR-199a-3p in cardiomyocytes
Source: Acta Biochim Biophys Sin (Shanghai). 2025 Feb 10;57(7):1164–74. doi: 10.3724/abbs.2024240 (PMC12422884; doi:10.3724/abbs.2024240)
Supplement: 465FigS1-5 [file 465FigS1-5.pdf]

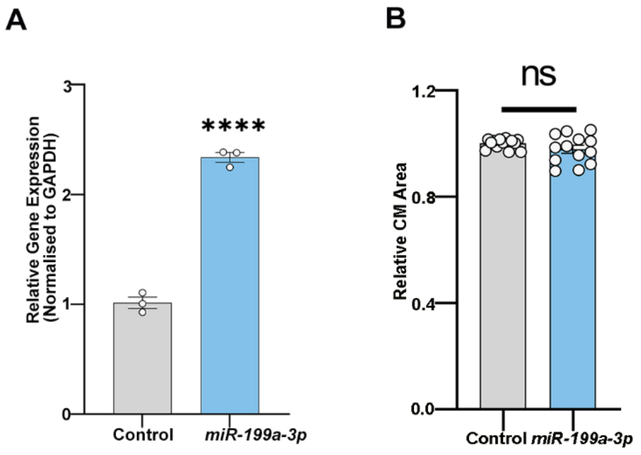

Figure S1. The efficiency of *miR-199a-3p* transfection and its effect on cardiomyocyte area of NRVCs.

(A) Relative expression of *miR-199a-3p* in NRVCs transfected with *miR-199a-3p* mimic and control. n = 3. (B) Quantification of relative cardiomyocyte area of NRVCs transfected with *miR-199a-3p* mimic and control. n is the well number, n=13. Data are expressed as mean ±SEM, ****$p < 0.0001$, ns, not significant.

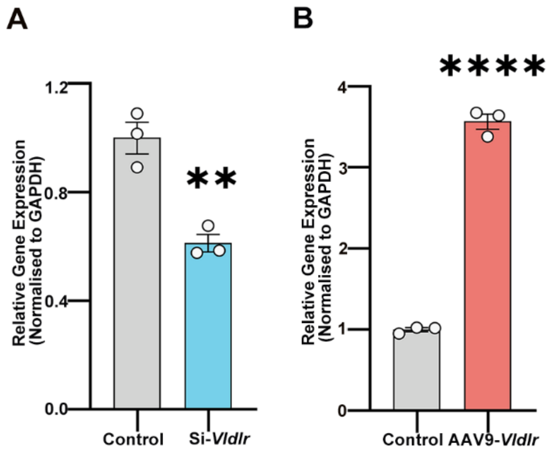

Figure S2. The efficiency of *Vldlr* knockdown and overexpression.

(A) qRT-PCR results of *Vldlr* expression in NRVCs transfected with si-*Vldlr* and control. n=3. (B) qRT-PCR results of *Vldlr* expression in NRVCs transduced with AAV9-*Vldlr* vs. control. n=3. Data are expressed as mean ±SEM, **$p < 0.01$, ****$p < 0.0001$.

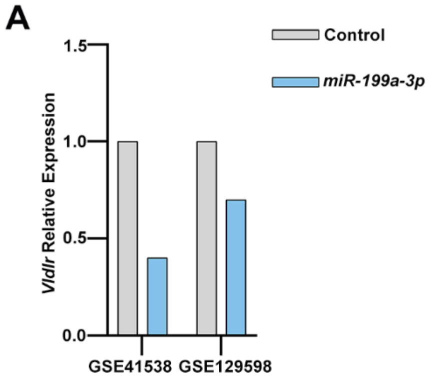

Figure S3. ***Vldlr*** **expression is downregulated in response to** ***miR-199a-3p*** **overexpression.**

(A) Relative mRNA expression levels of *Vldlr* in the neonatal mouse cardiomyocytes (GSE41538) and NRVCs (GSE129598) transfected with *miR-199a-3p* mimic vs. control determined by RNA-seq.

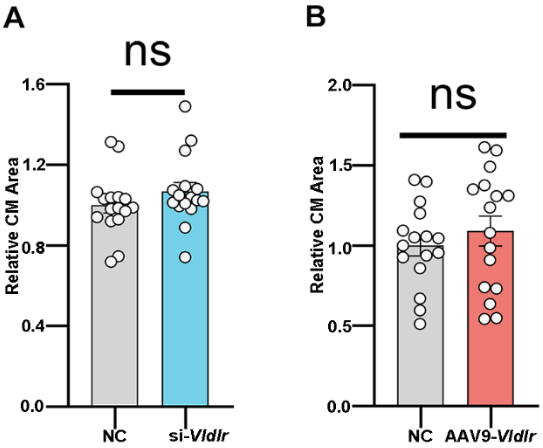

Figure S4. Cardiomyocyte area in NRVCs with ***Vldlr*** **knockdown and overexpression.**

(A) Quantification of relative cardiomyocyte area in NRVCs transfected with si-*Vldlr* and control. n is the well number, n=16. (B) Quantification of relative cardiomyocyte area in NRVCs transduced with AAV9-*Vldlr* and control. n is the image field, n=15. Data are expressed as mean ±SEM, ns, not significant.

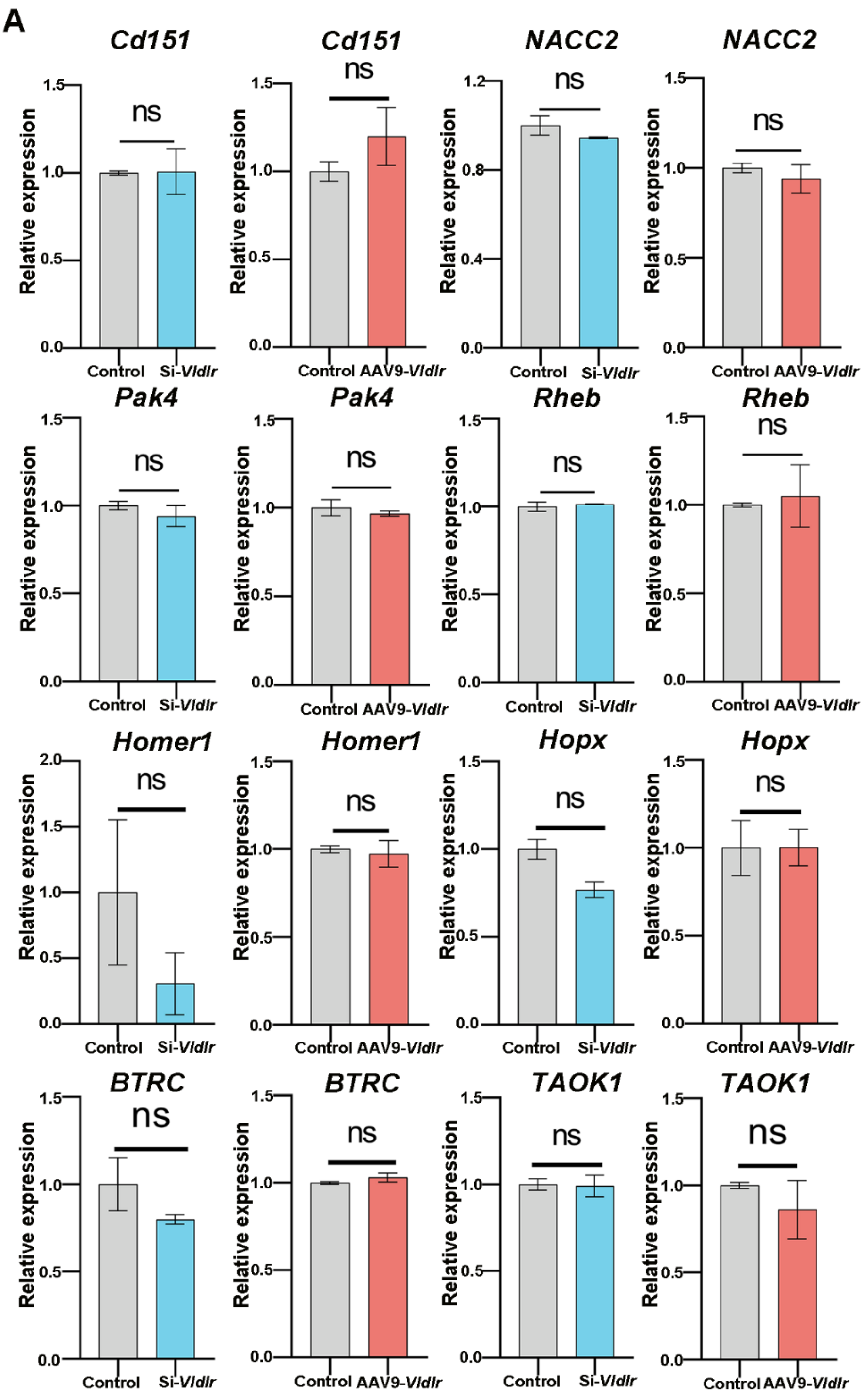

Figure S5. Effects of *Vldlr* knockdown and overexpression on *miR-199a-3p* target genes.

(A) mRNA expression levels of *miR-199a-3p* target genes (*Cd151*, *NACC2*, *Pak4*, *Rheb*, *Homer1, Hopx, BTRC,* and *TAOK1*) upon *Vldlr* knockdown and overexpression determined by RNA-seq. Data are expressed as mean ±SEM, ns, not significant.

Table S 1. Gene lists used in Figure 1E.
